# Aerobic Exercise Protects against Cardiotoxin-Induced Skeletal Muscle Injury in a DDAH1-Dependent Manner

**DOI:** 10.3390/antiox13091069

**Published:** 2024-09-01

**Authors:** Fei Feng, Kai Luo, Xinyi Yuan, Ting Lan, Siyu Wang, Xin Xu, Zhongbing Lu

**Affiliations:** 1 Sport and Health Science Department, Nanjing Sport Institute, Nanjing 210000, China; fengfei@sus.edu.cn; 2 College of Life Science, University of Chinese Academy of Sciences, Beijing 100049, China; luokai20@mails.ucas.ac.cn (K.L.); yuanxinyi21@mails.ucas.ac.cn (X.Y.); lanting21@mails.ucas.ac.cn (T.L.); wangsiyu23@mails.ucas.ac.cn (S.W.); 3 School of Exercise and Health, Shanghai University of Sport, Shanghai 200438, China

**Keywords:** exercise, DDAH1, cardiotoxin, skeletal muscle regeneration, oxidative stress

## Abstract

Dimethylarginine dimethylaminohydrolase 1 (DDAH1) is a critical enzyme that regulates nitric oxide (NO) signaling through the degradation of asymmetric dimethylarginine (ADMA). Previous studies have revealed a link between the beneficial effects of aerobic exercise and the upregulation of DDAH1 in bones and hearts. We previously reported that skeletal muscle DDAH1 plays a protective role in cardiotoxin (CTX)-induced skeletal muscle injury and regeneration. To determine the effects of aerobic exercise on CTX-induced skeletal muscle injury and the role of DDAH1 in this process, wild-type (WT) mice and skeletal muscle-specific *Ddah1*-knockout (*Ddah1*^MKO^) mice were subjected to swimming training for 8 weeks and then injected with CTX. In WT mice, swimming training for 8 weeks significantly promoted skeletal muscle regeneration and attenuated inflammation, oxidative stress, and apoptosis in the gastrocnemius (GA) muscle after CTX injection. These phenomena were associated with increases in the protein expression of PAX7, myogenin, MEF2A, eNOS, SOD2, and peroxiredoxin 5 and decreases in iNOS expression in GA muscles. Swimming training also decreased serum ADMA levels and increased serum nitrate/nitrite (NOx) levels and skeletal muscle DDAH1 expression. Interestingly, swimming training in *Ddah1*^MKO^ mice had no obvious effect on CTX-induced skeletal muscle injury or regeneration and did not repress the CTX-induced inflammatory response, superoxide generation, or apoptosis. In summary, our data suggest that DDAH1 is important for the protective effect of aerobic exercise on skeletal muscle injury and regeneration.

## 1. Introduction

Skeletal muscle injuries, including direct injuries (skeletal muscle lacerations, contusions, etc.), indirect injuries (for example, strains), and degenerative diseases (such as skeletal muscle atrophy), are frequently observed in daily life and sports [1,2,3,4]. These injuries may cause various degrees of impairment in daily life, social life, and physical activity [5] and subsequently negatively impact people’s quality of life [6].

The idea that “exercise is medicine” was developed by the American College of Sports Medicine and American Medical Association in 2007, which declared that physical activity can promote health and prevent diseases. Growing evidence confirms that exercise has similar effects as drugs do on improving human health, ameliorating a variety of chronic diseases, and preventing various skeletal muscle injuries [7,8,9,10]. For example, aerobic exercise can protect against sepsis-associated skeletal muscle injury [11], contractile-induced skeletal muscle damage [12], and doxorubicin-induced skeletal muscle atrophy [13,14]. Aerobic exercise can also affect skeletal muscle regeneration through regulating the activation [15,16] or self-renewal of satellite cells [17]. Therefore, further elucidation of the protective mechanism of aerobic exercise is conducive for skeletal muscle injury therapy.

Nitric oxide (NO) is a gas signal molecule produced immediately after skeletal muscle injury and plays an important role in muscle regeneration through modulating satellite cell activation [18,19]. In vivo, the production of NO is affected by the local concentration of asymmetric dimethylarginine (ADMA), an endogenous inhibitor of nitric oxide synthase (NOS). Dimethylarginine dimethylaminohydrolase-1 (DDAH1) is a critical enzyme for ADMA degradation [20], and genetic or pharmacological inhibition of DDAH1 attenuates NO signal transduction by promoting ADMA accumulation [20,21]. We recently showed that muscle DDAH1 plays a significant protective role in cardiotoxin (CTX)-induced skeletal muscle injury and regeneration [22]. It has also been demonstrated that aerobic exercise can upregulate the expression of DDAH1 in the heart and bone [23,24] and achieve corresponding protection. However, the role of skeletal muscle DDAH1 in the protective effect of aerobic exercise remains unclear.

In the present study, we first investigated the effect of aerobic exercise on CTX-induced skeletal muscle injury. We subsequently used muscle-specific *Ddah1*-deficient (*Ddah1*^MKO^) mice to determine whether skeletal muscle DDAH1 is involved in the beneficial effect of aerobic exercise in CTX-injected mice.

## 2. Materials and Methods

### 2.1. Reagents and Antibodies

CTX and wheat germ agglutinin (WGA) were obtained from MedChemExpress LLC (#HY-P1902A, Princeton, NJ, USA) and Biotium Inc. (#29022, Fremont, CA, USA), respectively. The TUNEL staining kit and total NO assay kit were obtained from Beyotime Institute of Biotechnology (#C1090 and #S0023, Shanghai, China). Dihydroethidium (DHE) was purchased from Sigma (#D7008, St. Louis, MO, USA). The ADMA ELISA kit and lactate dehydrogenase (LDH) assay kits were purchased from Bio-Techne Co., Ltd. (#NBP2–66728, Minneapolis, MN, USA) and the Nanjing Jiancheng Bioengineering Institute (#A020-2-2, Nanjing, China), respectively.

Antibodies against DDAH1, peroxiredoxin 3 (PRDX3), PRDX5, and β-tubulin were purchased from Signalway Antibody LLC (#37368, #38567, #38828 and #48659; Greenbelt, MD, USA). Antibodies against Bcl-2, Bax, eNOS and laminin were purchased from Abcam (#ab194583, #ab182733, #ab199956, and #ab7463, Cambridge, UK). Antibodies against F4/80, iNOS, and SOD2 were purchased from Wuhan Sanying Biology Technology Company (#28463-1-AP, #22226-1-AP, and #24127-1-AP; Wuhan, China). Antibodies against PAX7, MyoD, myogenin and MEF2A were obtained from Santa Cruz Biotechnology, Inc. (#sc-81648, #sc-32758, #sc-12732, and #sc-17785; Dallas, TX, USA). An antibody against MyH4 was obtained from Thermo Fisher Scientific, Inc. (#14-6503-82, Carlsbad, CA, USA).

### 2.2. Mouse Experiments

C57BL/6J mice and *Ddah1*^MKO^ mice aged 6–8 weeks were used. C57BL/6J mice were purchased from Beijing Vital River Laboratory Animal Technology Co., Ltd. (Beijing, China). As described previously [22], *Ddah1*^MKO^ mice were generated by crossing *Ddah1*^f/f^ mice and MCK-Cre mice, which were kindly provided by Professor YingJie Chen from the University of Minnesota and Professor Yan Zhang from Peking University, respectively.

The protocol for aerobic exercise followed the swimming scheme of mice in the *Resource Book for the Design of Animal Exercise Protocols*. In brief, after 1 week of adaptive training, the mice in the aerobic exercise group were subjected to 60 min of swimming every day for 8 weeks. The water temperature in the swimming tank was maintained at 32–35 °C. After swimming training, the mice were gently dried with a clean towel and placed in a cage with an insulation device to dry their fur at a constant temperature.

The mice were anesthetized with isoflurane, shaved with a small animal shaver, disinfected with 75% alcohol solution, and then subjected to a gastrocnemius (GA) muscle injection of CTX (100 μL, 10 μM). The mice in the control group were injected with an equal volume of saline. The mice were euthanized on Days 3 and 5 after a CTX injection, after which the GA muscle and serum were collected for subsequent analysis.

During the entire experimental period, the mice were kept in individually ventilated cages and had free access to commercial mouse chow and distilled water. The temperature in the animal room was maintained at 22~24 °C with a 12 h light/dark cycle. The protocols for the mouse experiments were approved by the University of Chinese Academy of Sciences Animal Care and Use Committee (UCAS-A-20221020).

### 2.3. Histopathological Analysis

GA muscle frozen sections (8 μm) were stained with hematoxylin and eosin (H&E), WGA, DHE, TUNEL kits, and antibodies against F4/80, PAX7, and laminin. Specifically, the WGA was used to label the skeletal muscle cell membrane, and then the cross-sectional area (CSA) was manually measured with NIH ImageJ software (Ver 1.51-java 8, Bethesda, MD, USA). A total of 5 mice per group were used for these experiments. During the histological analysis, group information was blind to the researcher to avoid bias.

### 2.4. Quantitative Real-Time PCR Analysis

Total RNA was extracted from mouse GA muscle tissue via TRIzol reagent. Reverse transcription was performed with a PrimeScript RT reagent kit (#RR036B, TaKaRa, Otsu, Japan). Quantitative real-time PCR was performed using a SYBR Premix Ex Taq™ II Kit (#RR820DS, TaKaRa). The relative quantification of gene expression was performed via the 2^−ΔΔCT^ method, and the data were normalized to 18S ribosomal RNA. The sequences of the primers used are shown in Table 1.

### 2.5. Western Blotting

Protein was extracted from GA muscle tissue via ice-cold RIPA lysis buffer (#P0013B, Beyotime, Shanghai, China) supplemented with 1% phenylmethylsulfonyl fluoride, as well as protease and phosphatase inhibitor cocktails from Roche (#4693124001 and #4906837001, Basel, Switzerland). The protocol for Western blotting was described in our previous report [22]. The dilutions of the primary antibodies and horseradish peroxidase-labeled secondary antibodies were 1:1000 and 1:10,000, respectively. The blots were visualized with a ChemiDoc™ XRS+ Gel Imaging System (Bio-Rad Laboratories, Inc., Hercules, CA, USA).

### 2.6. Statistical Analysis

All results are expressed as the means ± SEMs. GraphPad Prism 9 software (GraphPad Software Inc., San Diego, CA, USA) was used for the data analyses. Differences between groups for each variable were compared via either two-way analysis of variance or one-way analysis of variance after the post hoc Tukey test. *p* < 0.05 was considered statistically significant.

## 3. Results

### 3.1. Aerobic Exercise Improves CTX-Induced Skeletal Muscle Regeneration

**Figure 1:**
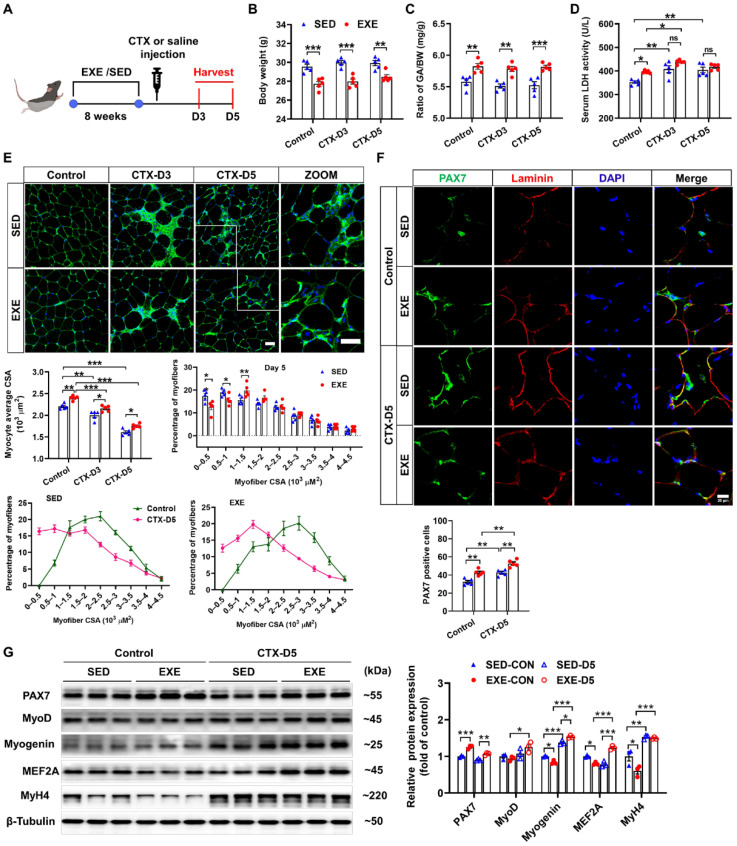
Aerobic exercise promotes skeletal muscle regeneration in cardiotoxin (CTX)-treated mice. (**A**) The experimental design of this study is shown in a schematic diagram. On Days 3 and 5 after CTX injection, the body weight (**B**), the GA weight-to-body weight (GA/BW) ratio (**C**), and the serum lactate dehydrogenase (LDH) level (**D**) were measured. (**E**) Representative cryosections from GA muscle were stained with wheat germ agglutinin (WGA). The myofiber average cross-sectional area (CSA) was measured via ImageJ. The distribution of GA myofiber size in control and CTX-treated mice is shown. Scale bar = 50 μm. (**F**) Cryosections of the GA muscle were stained with DAPI (blue) and antibodies specific for PAX7 (green) and laminin (red). Scale bar = 20 μm. (**G**) GA muscle was homogenized in RIPA buffer, and the lysates were examined via Western blotting. In Figure 1A–F, N = 5; in Figure 1G, N = 3; the values are expressed as the means ± SEMs. ns indicates not significant, * indicates *p* < 0.05, ** indicates *p* < 0.01, and *** indicates *p* < 0.001.

The group assignments and timeline of the experimental processes are illustrated in Figure 1A. C57BL/6J mice were randomly divided into exercised (EXE) and sedentary (SED) groups. After 8 weeks of swimming training, the mice were injected with CTX to induce skeletal muscle injury. GA muscle and serum were collected 3 and 5 days after CTX injection to determine the effects of exercise on muscle injury and regeneration [25]. CTX injection had no obvious effect on body weight or the ratio of GA weight-to-body weight (GA/BW) in either the SED or EXE groups. However, the mice in the EXE group presented lower body weights and higher GA/BW ratios than the mice in the SED group did (Figure 1B,C). LDH is a marker of skeletal muscle injury [26]. Under control conditions, the serum LDH levels were increased in the EXE group. After CTX injection, the serum LDH levels were increased in the SED group on Days 3 and 5. In the EXE group, the serum LDH levels were increased only on Day 3. However, there was no significant difference in the serum LDH levels between the EXE and SED groups (Figure 1D).

As revealed by WGA staining, CTX injection caused significant decreases in the average myofiber CSA in both groups. However, the mice in the EXE group presented larger myofiber CSA than did those in the SED group. On Day 5 after CTX injection, a leftward shift in myofiber size distribution was observed, and there were significant increases in the number of small muscle fibers (0~0.5 × 10^3^ μm^2^), which are regarded as newly formed muscle fibers, in both groups. Compared with the mice in the SED group, the mice in the EXE group had fewer muscle fibers of small size (0~0.5 × 10^3^ μm^2^ and 0.5~1 × 10^3^ μm^2^) and more muscle fibers of medium size (1~1.5 × 10^3^ μm^2^) (Figure 1E).

Next, PAX7/laminin immunofluorescence staining was performed on GA muscle frozen sections to determine the effect of aerobic exercise on SC activation. Under control conditions, more PAX7-positive cells were found in the EXE group. CTX injection significantly increased the number of PAX7-positive cells in both groups, whereas more PAX7-positive cells were detected in the EXE group than in the SED group (Figure 1F). Western blot analysis revealed that PAX7 protein expression was greater, whereas the protein expression of myogenin, MEF2A, and MyH4 was lower in the GA muscle tissue of the EXE group than in that of the SED group under control conditions. CTX injection increased the protein expression of myogenin and MyH4 in the GA muscles from both groups. However, the CTX-induced upregulation of MyoD and MEF2A was observed only in the EXE group. In addition, the expression of myogenin and MEF was greater in the GA muscle from the EXE group than in that from the SED group (Figure 1G).

### 3.2. Aerobic Exercise Attenuated the Inflammatory Response after CTX Treatment

**Figure 2:**
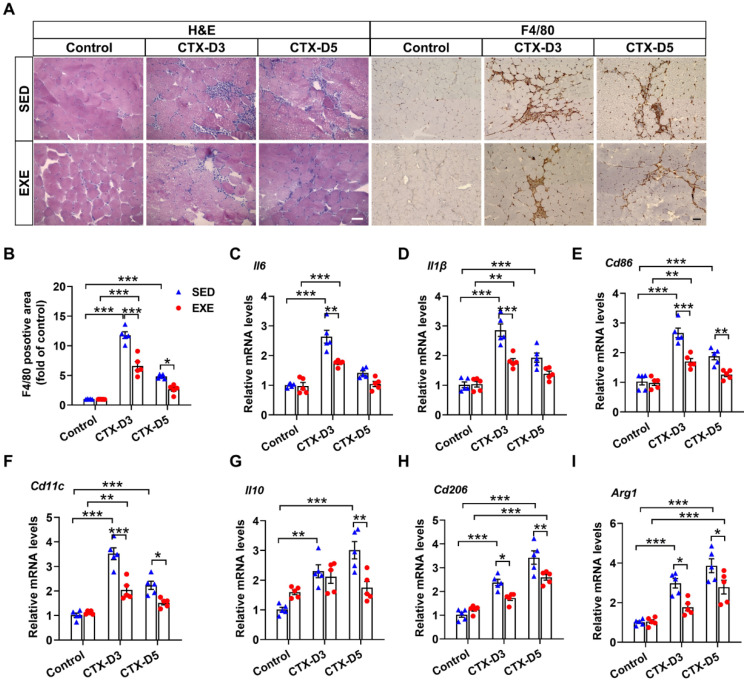
Aerobic exercise attenuated the CTX-induced skeletal muscle inflammatory response. (**A**) Representative cryosections of GA muscle were stained with hematoxylin and eosin (H&E) and an antibody specific for F4/80 (brown staining). Scale bar = 50 μm. (**B**) F4/80-positive cell numbers were quantified via ImageJ. (**C**–**I**) The mRNA levels of M1 and M2 macrophage-specific markers in each group were measured on Days 3 and 5 after CTX injection. In Figure 2A–I, N = 5; the values are expressed as the mean ± SEM. * indicates *p* < 0.05, ** indicates *p* < 0.01, and *** indicates *p* < 0.001.

The persistence of inflammation reportedly reduces the regeneration efficiency of skeletal muscle [27,28]. H&E staining revealed less inflammatory cell infiltration in the GA muscle from the EXE group than in that from the SED group on Days 3 and 5 after CTX injection (Figure 2A). F4/80 is highly expressed on various macrophages and can be used as a specific marker for mouse macrophages [29]. Immunohistochemical staining with an F4/80 antibody revealed that mice from the EXE group also exhibited less macrophage infiltration in the GA muscle on Days 3 and 5 (Figure 2A,B).

It is well known that macrophages can be divided into M1 proinflammatory macrophages and M2 anti-inflammatory macrophages. The balance of M1/M2 macrophages has profound effects on skeletal muscle regeneration in the later stage [30]. To further determine the effects of aerobic exercise on the skeletal muscle inflammatory response and macrophage recruitment, the expression of specific markers of M1 and M2 macrophages was measured. On Day 3, CTX injection caused more significant increases in the mRNA levels of M1 macrophage-specific markers (*interleukin-6* (*Il6*), *Il1β*, *Cd86*, and *Cd11c*) in the SED group than in the EXE group. CTX injection also increased the mRNA levels of M2 macrophage-specific markers (*Il10*, *Cd206*, and *Arg1*) in the SED group but not in the EXE group, and the mRNA levels of *Cd206* and *Arg1* were lower in the EXE group than in the SED group. On Day 5, CTX injection had no obvious effect on the mRNA levels of M1 macrophage-specific markers in the EXE group but increased the mRNA levels of *Cd86* and *Cd11c* in the SED group. CTX injection also caused greater increases in the mRNA levels of *Il10*, *Cd206*, and *Arg1* in the SED group than in the EXE group (Figure 2C–F). Together, these results suggest that aerobic exercise may improve skeletal muscle injury by attenuating the inflammatory response and the recruitment of proinflammatory and anti-inflammatory macrophages.

### 3.3. Aerobic Exercise Reduced the Apoptosis and Oxidative Stress Induced by CTX

**Figure 3:**
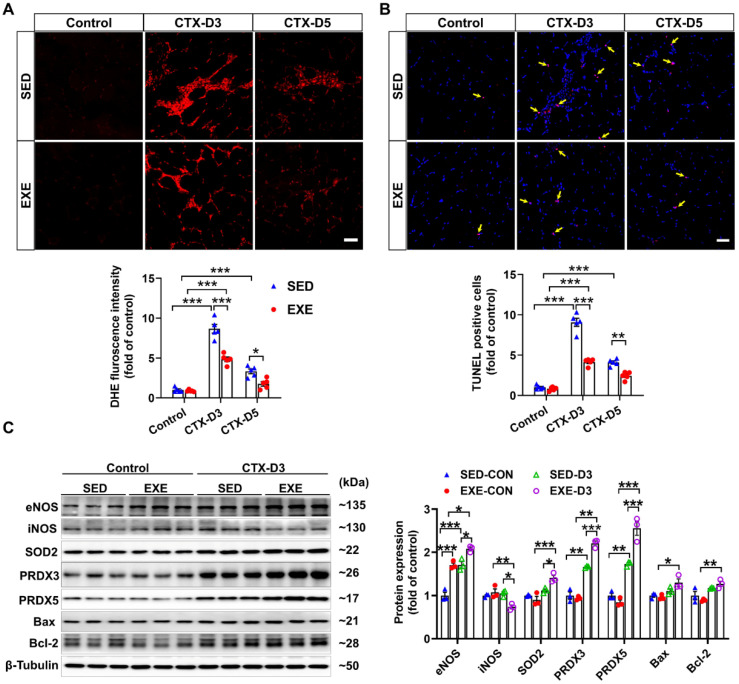
Aerobic exercise ameliorated CTX-induced apoptosis and oxidative stress in GA muscle. (**A**) Representative frozen GA muscle sections from each group were stained with DHE, and the relative fluorescence intensity was quantified; scale bar = 50 μm. (**B**) Representative frozen skeletal muscle sections from each group were subjected to TUNEL, and the number of apoptotic cells was quantified; scale bar = 50 μm. (**C**) GA muscle lysates were subjected to Western blot analysis. In Figure 3A,B, N = 5; in Figure 3C, N = 3; the values are expressed as the means ± SEMs. * indicates *p* < 0.05, ** indicates *p* < 0.01, and *** indicates *p* < 0.001.

Previous reports have shown that oxidative stress, especially excessive superoxide production, can delay skeletal muscle regeneration [31] and that the number of apoptotic cells in the muscle is strongly associated with the severity of skeletal muscle injury [32]. To determine the effects of aerobic exercise on CTX-induced oxidative stress and apoptosis, frozen GA muscle sections were stained with DHE and TUNEL kits, respectively. On Day 3, CTX injection caused fewer increases in superoxide levels and apoptotic cell numbers in the GA muscle of the EXE group than in the SED group. On Day 5, the CTX-induced increases in superoxide production and apoptotic cell numbers were found only in the GA muscle of the mice in the SED group. In addition, the superoxide levels and number of apoptotic cells in the GA muscle were lower in the EXE group than in the SED group (Figure 3A,B).

To investigate the potential mechanism by which aerobic exercise attenuates CTX-induced oxidative stress and apoptosis, we performed Western blot analysis to determine the changes in the protein expression of NOS, antioxidant enzymes, and pro- or antiapoptotic proteins. Under control conditions, swimming training increased the protein expression of eNOS in the GA muscle. In the SED group, CTX injection increased the protein expression of eNOS, PRDX3, and PRDX5 but had no obvious effect on the expression of iNOS, SOD2, Bcl-2, or Bax on Day 3. However, CTX caused a significant decrease in iNOS expression and increases in the protein expression of eNOS, SOD2, PRDX5, Bcl-2, and Bax in the GA muscle of the EXE group. Compared with those in the SED group, the protein expression levels of eNOS, SOD2, PRDX5, Bcl-2, and Bax were greater, whereas the protein expression level of iNOS was lower in the GA muscle of the mice in the EXE group (Figure 3C). These results suggest that aerobic exercise may improve skeletal muscle injury by increasing the expression of eNOS, antioxidant enzymes, and Bcl-2 to reduce oxidative stress and cell apoptosis.

### 3.4. The Protective Effects of Aerobic Exercise on CTX-Induced Skeletal Muscle Injury Were Diminished in Skeletal Muscle-Specific Ddah1 Knockout Mice

**Figure 4:**
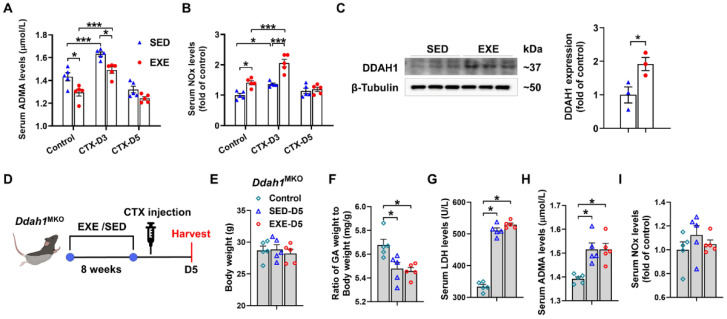
DDAH1 in skeletal muscle is involved in the protective effect of aerobic exercise on CTX-induced muscle injury. Serum asymmetric dimethylarginine (ADMA) (**A**) and nitrate/nitrite (NOx) (**B**) levels were measured on Days 3 and 5 after CTX injection. (**C**) DDAH1 expression in the GA muscle was examined via Western blotting. (**D**) The experimental process for *Ddah1*^MKO^ mice is shown in a schematic diagram. On Day 5 after CTX injection, the body weight (**E**), the GA weight-to-body weight ratio (**F**) and the serum levels of LDH (**G**), ADMA (**H**) and NOx (**I**) were measured. In Figure 4A,B and E–I, N = 5; in Figure 4C, N = 3; the values are expressed as the means ± SEMs. * indicates *p* < 0.05, *** indicates *p* < 0.001.

To determine whether DDAH1 is involved in the protective effect of aerobic exercise, we examined the serum levels of ADMA and nitrate/nitrite (NOx) in both the EXE and SED groups. Under control conditions, serum ADMA levels were decreased, whereas serum NOx levels were increased in the EXE group. On Day 3, CTX injection caused less increases in serum ADMA levels but more increases in serum NOx levels in the EXE group than in the SED group. However, there was no significant difference in the serum ADMA and NOx levels between the SED and EXE groups on Day 5 (Figure 4A,B). Western blot analysis revealed that swimming training for 8 weeks significantly increased DDAH1 expression in the GA muscle (Figure 4C). The age-matched *Ddah1*^MKO^ mice were then randomly divided into three groups: the control, SED, and EXE groups. The mice in the EXE group were subjected to swimming training for 8 weeks and injected with CTX (Figure 4D). CTX injection did not affect the body weight and caused similar decreases in the GA weight-to-body weight ratio and increases in the serum LDH and ADMA levels in the SED and EXE groups (Figure 4E–H). The serum NOx levels were unaffected by CTX injection on Day 5, and there was no difference between the SED and EXE groups (Figure 4I).

### 3.5. Aerobic Exercise Had No Obvious Effect on CTX-Induced Skeletal Muscle Regeneration in Ddah1^MKO^ Mice

**Figure 5:**
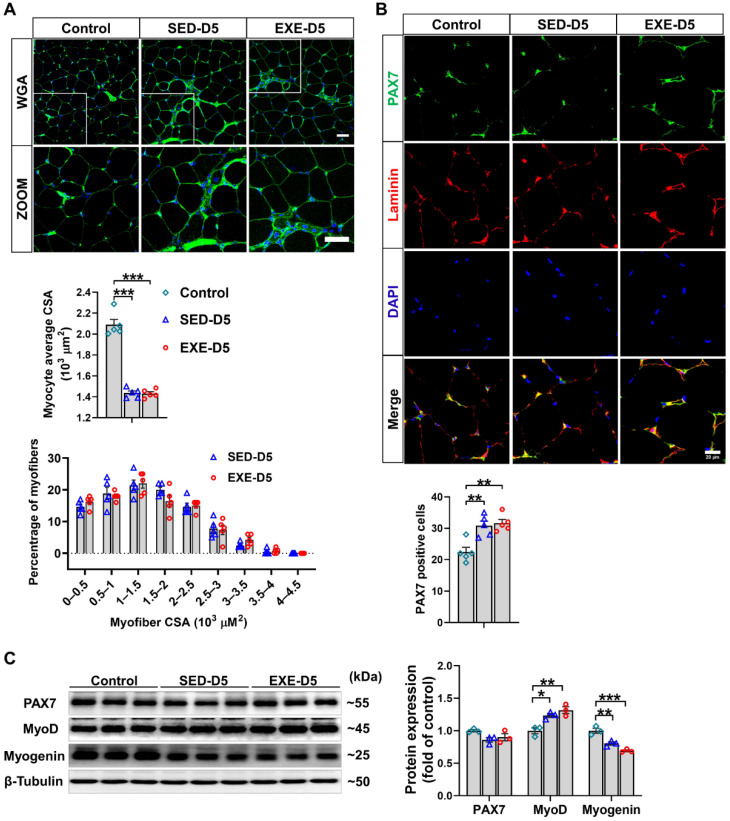
Aerobic exercise did not promote CTX-induced skeletal muscle regeneration in *Ddah1*^MKO^ mice. (**A**) Cryosections from the GA muscle in each group were stained with WGA; scale bars = 50 μm. The average myofiber CSA was measured via ImageJ, and the GA myofiber size distributions in control and CTX–treated mice are shown. (**B**) Cryosections from control and CTX–treated GA muscle were subjected to immunofluorescence, and SCs were labeled with both PAX7 (green) and DAPI (blue). Scale bar = 20 μm. PAX7-positive cells are shown, and each mouse skeletal muscle tissue section was randomly photographed in 10 visual fields, after which statistical analysis was performed. (**C**) GA muscle was homogenized in RIPA buffer, and the lysates were examined via Western blotting. In Figure 5A–E, N = 5; in Figure 5F, N = 3; the values are expressed as the means ± SEMs. * indicates *p* < 0.05, ** indicates *p* < 0.01, and *** indicates *p* < 0.001.

WGA staining of the frozen sections of GA muscle revealed that CTX injection in *Ddah1*^MKO^ mice resulted in similar decreases in the average muscle fiber CSA in the SED and EXE groups. There was also no difference in the muscle fiber size distribution between the EXE and SED groups, especially the small muscle fibers (Figure 5A). Immunofluorescence staining with PAX7/laminin-specific antibodies revealed that the CTX-induced increases in PAX7-positive cell numbers were almost the same in the SED and EXE groups (Figure 5B). To further determine the effect of aerobic exercise on CTX-induced skeletal muscle regeneration in *Ddah1*^MKO^ mice, the protein expression of skeletal muscle regeneration-related proteins was examined via Western blotting. CTX injection did not affect PAX7 expression but increased MyoD expression and decreased myogenin in both groups. The expression of PAX7, MyoD, and myogenin was nearly the same in the GA muscle of *Ddah1*^MKO^ from the SED and EXE groups (Figure 5C). Together, the above results suggested that aerobic exercise in *Ddah1*^MKO^ mice had no obvious effect on muscle regeneration after CTX injection.

### 3.6. Aerobic Exercise Did Not Affect CTX-Induced Oxidative Stress or Apoptosis in the GA Muscle of Ddah1^MKO^ Mice

**Figure 6:**
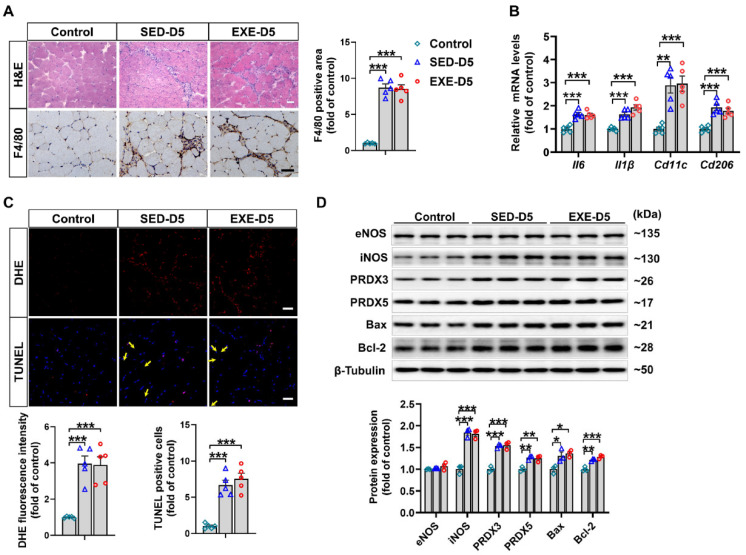
Aerobic exercise had no effect on CTX-induced inflammation, oxidative stress or apoptosis in the GA muscle of *Ddah1*^MKO^ mice. (**A**) Representative frozen GA muscle sections were stained with H&E and an antibody specific for F4/80 (brown staining); scale bars = 50 μm. The number of F4/80-positive cells was quantified. (**B**) The mRNA levels of inflammatory genes were measured via qPCR. (**C**) Representative frozen GA muscle sections were stained with DHE and TUNEL kits. The relative fluorescence intensity and number of apoptotic cells were quantified, scale bars = 50 μm. (**D**) GA muscle lysates were examined via Western blotting. In Figure 6A–C, N = 5. In Figure 6D, N = 3; the values are expressed as the means ± SEMs, * indicates *p* < 0.05, ** indicates *p* < 0.01, and *** indicates *p* < 0.001.

As shown by H&E staining, there was a similar degree of inflammatory cell infiltration in the GA muscle of *Ddah1*^MKO^ mice in the SED and EXE groups after CTX injection. Immunohistochemical staining with an anti-F4/80 antibody revealed significant infiltration of macrophages into the GA muscle of *Ddah1*^MKO^ mice. However, the F4/80-positive area was almost the same in the two groups (Figure 6A). In addition, the mRNA levels of *Il6*, *Il1β*, *Cd11c*, and *Cd206* in the GA muscle of *Ddah1*^MKO^ mice in both the SED and EXE groups were notably increased in response to CTX injection, but there was no significant difference in the upregulation of those genes between the two groups (Figure 6B).

We then examined the effects of aerobic exercise on CTX-induced oxidative stress and apoptosis in *Ddah1*^MKO^ mice. As revealed by DHE and TUNEL staining, superoxide levels and the number of apoptotic cells were significantly increased in the GA muscle CTX-injected mice. However, the increases in superoxide levels and apoptotic cells did not differ between the SED and EXE groups (Figure 6C). After CTX injection, the protein expression of iNOS, PRDX3, PRDX5, Bax, and Bcl-2 was clearly increased in the GA muscle of *Ddah1*^MKO^ mice in the SED and EXE groups. The changes in the expression of these proteins were nearly the same between the two groups (Figure 6D).

## 4. Discussion

The present study has two major findings. First, we demonstrated that aerobic exercise can promote skeletal muscle regeneration after CTX injection, which is associated with attenuated inflammation, oxidative stress, and apoptosis. Second, aerobic exercise can increase muscle DDAH1 expression, and skeletal muscle-specific *Ddah1* knockout diminished the protective effect of aerobic exercise on CTX-induced skeletal muscle injury.

The beneficial effects of exercise were first proposed in 1968 by Peter Karpovich [33]. Growing studies have proved that exercise can affect the occurrence and progression of human cardiovascular disease [34], type 2 diabetes [35], non-alcoholic fatty liver disease [36], and Parkinson’s disease [37] and play a role in prevention and rehabilitation. In particular, sarcopenia in the elderly is induced by the reduction of anabolic stimulation due to lack of physical activity [38,39], while aerobic exercise can alleviate the decline of muscle mass and loss of function by promoting the anabolism of muscle protein in the elderly [40,41]. Other studies have shown that protein and vitamin D supplements combined with exercise can also improve sarcopenia [42] and osteoporosis in postmenopausal women [43].

The beneficial effect of aerobic exercise on skeletal muscle-related diseases has long been acknowledged. For example, aerobic exercise can effectively reverse the decline in endurance capacity and alleviate skeletal muscle atrophy in aged mice [44]. Exercise can also protect against doxorubicin-induced skeletal muscle atrophy [45]. Growing evidence also suggests that aerobic exercise enhances skeletal muscle regeneration by affecting the activation, proliferation, differentiation, and fusion of satellite cells [46]. In the present study, we also showed that the mice in the aerobic exercise group had greater skeletal muscle regeneration efficiency after CTX-induced injury, as evidenced by the increased average CSA of muscle fibers and the greater proportion of medium-sized muscle fibers (1~1.5 × 10^3^ μm^2^) in the GA muscle of the EXE group. PAX7 and MyoD are expressed in activated satellite cells [47], whereas myogenin and MEF2A are expressed in differentiated satellite cells [48] and help skeletal muscle myoblasts fuse [49]. Here, we demonstrated that GA muscle from the EXE group had more PAX7-positive cells and higher expression of PAX7, MyoD, myogenin, and MEF2A on Day 5 after CTX injection, suggesting that aerobic exercise caused significant increases in the numbers of activated and differentiated satellite cells, which are required for skeletal muscle regeneration. A similar effect of aerobic exercise on satellite cells has also been observed in aged mice [50].

Aerobic exercise may also affect skeletal muscle regeneration through modulating the immune response. During the process of skeletal muscle regeneration, M1 macrophages transition to the M2 subtype [51,52,53]. The infiltrated M1 macrophages in the early stage of muscle injury can clear necrotic skeletal muscle fragments and promote satellite cell activation and proliferation. After reaching peak numbers 2–3 days following injury, M1 macrophages transform into M2 macrophages to aid in the repression of inflammation and differentiation of satellite cells. Delaying or halting the timely transition of M1 to M2 macrophages is detrimental to skeletal muscle regeneration [52,54,55,56,57]. Here, we showed that aerobic exercise significantly reduced the infiltration of F4/80-positive macrophages after CTX injection. Moreover, aerobic exercise not only decreased the expression of M1 macrophage marker genes but also decreased the expression of M2 macrophage marker genes. It is likely that aerobic exercise may promote skeletal muscle regeneration by reducing overall macrophage infiltration and repressing the inflammatory response.

Aerobic exercise can regulate the muscular antioxidant system to maintain redox balance under stress conditions [58]. Aerobic exercise can increase the antioxidant capacity of the diaphragm [59], thereby preventing contraction-induced oxidative stress [59] and protecting against cigarette smoke-induced oxidative damage [60]. Here, aerobic exercise significantly decreased superoxide levels and increased the protein expression of SOD2 and PRDX5 in injured GA muscle. It has been well established that aerobic exercise exerts antiapoptotic effects under some stress conditions [61,62,63]. A recent report showed that aerobic exercise alleviates skeletal muscle apoptosis in aged rats by regulating the Trx system [64]. We also showed that aerobic exercise significantly attenuated apoptosis in the GA muscle after CTX injection. Considering that both oxidative stress and apoptosis are important obstacles for muscle regeneration, it is possible that aerobic exercise may also promote skeletal muscle regeneration through the inhibition of oxidative stress and apoptosis.

Consistent with previous observations that aerobic exercise training is associated with increased NO signaling [65], we also found higher serum NOx levels and lower serum ADMA levels in the EXE group than in the SED group, which was associated with significant increases in skeletal muscle DDAH1 expression. Exercise-induced DDAH1 upregulation has also been reported in the heart [23] and bone [24]. We consistently reported that DDAH1 exerts protective effects in different models, including high-fat diet-induced hepatic steatosis [66], PM2.5-induced lung injury [67], and pressure-overloaded hearts [68]. More interestingly, we recently demonstrated that DDAH1 was upregulated in the GA muscle of CTX-injected mice and that *Ddah1*^MKO^ mice presented more severe skeletal muscle injury and delayed regeneration after CTX injection. In addition, muscle-specific deletion of *Ddah1* exacerbated the CTX-induced inflammatory response, oxidative stress, and apoptosis [22]. Here, we demonstrated that aerobic exercise in *Ddah1*^MKO^ mice had no obvious effect on skeletal muscle regeneration after CTX injection. The attenuated effects of aerobic exercise on inflammation, oxidative stress, and apoptosis were almost completely diminished in *Ddah1*^MKO^ mice after CTX injection. Therefore, our results provide direct evidence that DDAH1 is important for the protective effect of aerobic exercise on skeletal muscle regeneration in CTX-injected mice.

## 5. Conclusions

In summary, our data suggest that aerobic exercise for 8 weeks effectively protects against CTX-induced skeletal muscle injury and promotes muscle regeneration by upregulating skeletal muscle DDAH1 expression, thereby inhibiting skeletal muscle inflammation, oxidative stress, and apoptosis.

## Figures and Tables

**Table 1:** The primers used in Quantitative real-time PCR.

Genes	Gene ID	Primers	Sequence
*Il6*	16193	Forward	5′-AACGATGATGCACTTGCAGA-3′
		Reverse	5′-TGGTACTCCAGAAGACCAGAGG-3′
*Il1β*	16176	Forward	5′-AGGTCAAAGGTTTGGAAGCA-3′
		Reverse	5′-TGAAGCAGCTATGGCAACTG-3′
*Cd86*	12524	Forward	5′-AGTGATCGCCAACTTCAGTGAACC-3′
		Reverse	5′-GGTGACCTTGCTTAGACGTGCAG-3′
*Cd11c*	16411	Forward	5′-CTGGATAGCCTTTCTTCTGCTG-3′
		Reverse	5′-GCACACTGTGTCCGAACTCA-3′
*Il10*	16153	Forward	5′-CTTACTGACTGGCATGAGGATCA-3′
		Reverse	5′-GCAGCTCTAGGAGCATGTGG-3′
*Cd206*	17533	Forward	5′-CTCTGTTCAGCTATTGGACGC-3′
		Reverse	5′-TGGCACTCCCAAACATAATTTGA-3′
*Arg1*	11846	Forward	5′-GAACACGGCAGTGGCTTTAAC-3′
		Reverse	5′-TGCTTAGCTCTGTCTGCTTTGC-3′
*18s*	19791	Forward	5′-TTCTGGCCAACGGTCTAGACAAC-3′
		Reverse	5′-CCAGTGGTCTTGGTGTGCTGA-3′

## Data Availability

The data presented in this study are available upon reasonable request from the corresponding author.
